# Application of Immune Infiltration Signature and Machine Learning Model in the Differential Diagnosis and Prognosis of Bone-Related Malignancies

**DOI:** 10.3389/fcell.2021.630355

**Published:** 2021-04-15

**Authors:** Guo-Qi Li, Yi-Kai Wang, Hao Zhou, Lin-Guang Jin, Chun-Yu Wang, Mugahed Albahde, Yan Wu, Heng-Yuan Li, Wen-Kan Zhang, Bing-Hao Li, Zhao-Ming Ye

**Affiliations:** ^1^Department of Orthopedics, Musculoskeletal Tumor Center, The Second Affiliated Hospital, School of Medicine, Zhejiang University, Hangzhou, China; ^2^Orthopedics Research Institute of Zhejiang University, Hangzhou, China; ^3^Key Laboratory of Motor System Disease Research and Precision Therapy of Zhejiang Province, Hangzhou, China; ^4^Department of Hepatobiliary and Pancreatic Surgery, School of Medicine, The Second Affiliated Hospital, Zhejiang University, Hangzhou, China

**Keywords:** osteosarcoma, Ewing’s sarcoma, multiple myeloma, bone metastases, immune microenvironment, ssGSEA, random forest

## Abstract

Bone-related malignancies, such as osteosarcoma, Ewing’s sarcoma, multiple myeloma, and cancer bone metastases have similar histological context, but they are distinct in origin and biological behavior. We hypothesize that a distinct immune infiltrative microenvironment exists in these four most common malignant bone-associated tumors and can be used for tumor diagnosis and patient prognosis. After sample cleaning, data integration, and batch effect removal, we used 22 publicly available datasets to draw out the tumor immune microenvironment using the ssGSEA algorithm. The diagnostic model was developed using the random forest. Further statistical analysis of the immune microenvironment and clinical data of patients with osteosarcoma and Ewing’s sarcoma was carried out. The results suggested significant differences in the microenvironment of bone-related tumors, and the diagnostic accuracy of the model was higher than 97%. Also, high infiltration of multiple immune cells in Ewing’s sarcoma was suggestive of poor patient prognosis. Meanwhile, increased infiltration of macrophages and B cells suggested a better prognosis for patients with osteosarcoma, and effector memory CD8 T cells and type 2 T helper cells correlated with patients’ chemotherapy responsiveness and tumor metastasis. Our study revealed that the random forest diagnostic model based on immune infiltration can accurately perform the differential diagnosis of bone-related malignancies. The immune microenvironment of osteosarcoma and Ewing’s sarcoma has an important impact on patient prognosis. Suppressing the highly inflammatory environment of Ewing’s sarcoma and promoting macrophage and B cell infiltration may have good potential to be a novel adjuvant treatment option for osteosarcoma and Ewing’s sarcoma.

## Introduction

The tumor immune microenvironment shapes tumorigenesis and development (Wu and Dai, 2017) and the diagnosis, treatment, and prognosis of tumor patients (Chen and Mellman, 2013; Clara et al., 2020). Tumor immune infiltration characteristics can be used to classify tumors into immune subtypes and potentially influence the choice of treatment options for patients (Angell et al., 2020). Bone tissue plays a major row in immune microenvironmental homeostasis, the bone microenvironment is an ideal fertile soil for both primary and secondary tumors to seed. Many malignant tumors occur in the bone, including osteosarcoma (OS), Ewing’s sarcoma (EW), and multiple myeloma (MM), with an incident rate of 4.0–5.4 per million, 1.5 per million (Ottaviani and Jaffe, 2009; Grünewald et al., 2018), and 43 per million (Kyle et al., 2004). Meanwhile, cancer metastasis remains the major cause of cancer-related mortality, and multiple cancers predispose to bone metastasis; approximately 70% of breast and prostate cancers are associated with bone metastases (Macedo et al., 2017; Fornetti et al., 2018; Hernandez et al., 2018).

In current clinical practice, pathology, immunohistochemistry, and radiography are essential to establish diagnosis and differential diagnosis for bone-related malignant diseases, yet difficulties often accompany the diagnostic process. By far, studies have indicated that there is a high rate of misdiagnosis and missed diagnosis of OS based on imaging and medical history, especially in elderly patients, with an incident rate of 23–43% (Chen and Mellman, 2013; Wu and Dai, 2017; Clara et al., 2020). In EW, Wurtz et al. reported that the average delay in diagnosis of EW was about 10 months (Wurtz et al., 1999), and Widhe et al. found a misdiagnosis rate of 80.77% in EW (Wurtz et al., 1999; Widhe et al., 2007). It is also often difficult to diagnose bone metastatic carcinoma with an unknown primary focus, and confirmation of diagnosis usually requires a combination of PET-CT and other tests (Guzik and Barañska, 2013). The immune infiltration characteristics of different tumors may provide an important tool for the differential diagnosis of tumors. Existing studies suggest that MM is a malignant tumor with a predominance of B cells (Kastrinakis et al., 2000). In contrast, cancer bone metastasis is usually associated with extensive immunosuppression (Liu and Cao, 2016), EW presents extensive inflammatory features (Durbin et al., 1998; Jordanov et al., 2009; Huang et al., 2013), yet the main components of OS are osteoblasts and osteoclasts, which originate mainly from the myeloid cell system, and macrophages make up the highest percentage of its immune microenvironment (Zhang et al., 2020). Besides, the immune microenvironment of bone-associated malignancies also influences the biological behavior of the tumor and the patient’s prognosis. B cells, MDSC, and other cells in the MM microenvironment can facilitate its migration and proliferation by secreting a variety of cytokines (Kastrinakis et al., 2000; Kumar et al., 2017); targeted intervention of tumor microenvironment B cells has significant efficacy in MM treatment (Shah et al., 2020). For OS, clinical studies have shown that patients with increased infiltration of CD163-positive macrophages have a better prognosis and that stimulation of monocyte and macrophage infiltration in OS by mifamurtide can prolong the disease-free survival of patients (Buddingh et al., 2011; Gomez-Brouchet et al., 2017). In addition, the immune microenvironment may also contribute to the cancer bone metastasis process (Janiszewska et al., 2019). In breast cancer, bone metastases can be significantly suppressed by restoring the inherent IFN signaling pathway in tumor cells and activating both the innate and acquired immune responses (Bidwell et al., 2012). Therefore, the use of the immune microenvironment in bone-associated tumors for diagnosis and prognosis has significant clinical potential.

There are various methods to study the bone immune microenvironment, such as single-cell sequencing, CYTOF, and computer-assisted algorithms such as CIBERSORT and single-sample gene set enrichment analysis (ssGSEA). Among them, ssGSEA’s unique non-parametric algorithm enables us to perform immuno-infiltration microenvironmental analysis across batches of independent samples and makes full use of the existing microarray and sequencing results. A large number of bone-related tumor microarrays and sequencing datasets have been published, including MM, OS, EW, and prostate cancer bone metastases (BM), making the development of immune infiltration-based differential diagnostic models very cost-effective and with great potential for application. In addition, the rapid development of machine learning algorithms has provided a unique opportunity for molecular-based cancer differential diagnosis. However, no studies to date have examined whether immune microenvironment profiles can be used for the differential diagnosis of bone-related tumors and used for prognostic analysis across datasets.

We hypothesize that immune microenvironment profiles can be used in the differential diagnosis of bone-associated tumors and have a significant prognosis for patients with bone-associated malignancies. Therefore, this study intends to use the ssGSEA algorithm for the immune microenvironment profiling of four tumors, combined with random forest machine learning methods to develop a bone-related tumor differential diagnosis model. On this basis, we will explore the influence of the immune microenvironment on the prognosis of tumor patients. It will provide new insight and theoretical basis for the diagnosis and treatment of related tumors.

## Results

### Immune Infiltration Profiling

**FIGURE 1:**
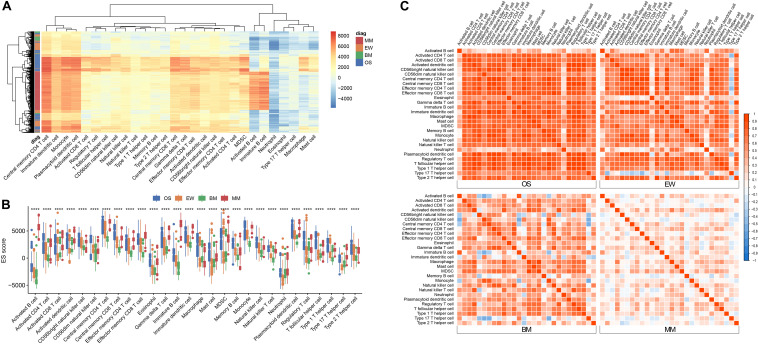
Immune infiltration signature across four diseases. **(A)** Heatmap of ES score across all samples. **(B)** Boxplot of ES score grouped by tumor type; asterisk (*) denotes statistical significance examined by ANOVA test ****p < 0.0001. **(C)** Correlation matrix of ES score among the 28 cell types within each disease; red represents positive correlation, and blue represents negative correlation. MM, multiple myeloma; EW, Ewing’s sarcoma; BM, prostate cancer bone metastases; OS, osteosarcoma.

After downloading the corresponding datasets and standardizing the data, we removed the cross-platform batch effects *via* the ComBat method in R. Thereafter, the immune enrichment scores (ES score) of 28 immune cells in each of the 1,459 samples were obtained using the ssGSEA algorithm. Unsupervised clustering was performed using Euclidean distances (Figure 1A); the result showed the 28 types of immune cells that are prevalent in all four types of tumors, and similar enrichment profiles can be observed within and across tumor types.

B cells are known to predominate in MM and can be used as a reliable positive control for cross-tumor comparisons. To clarify the differences in the infiltration of each immune cell in different tumors, we used ANOVA for comparison (Figure 1B). As predicted, the ES score of B cells was significantly elevated in MM, but not in OS, EW, or BM. CD56dim NK cells, macrophages, and Treg cells are mostly enriched in OS. BM has relatively low infiltration of various T cells, B cells, and plasmacytoid dendritic cells, which is consistent with the prevailing knowledge of the suppressive immune microenvironment in bone metastatic cancer.

**FIGURE 2:**
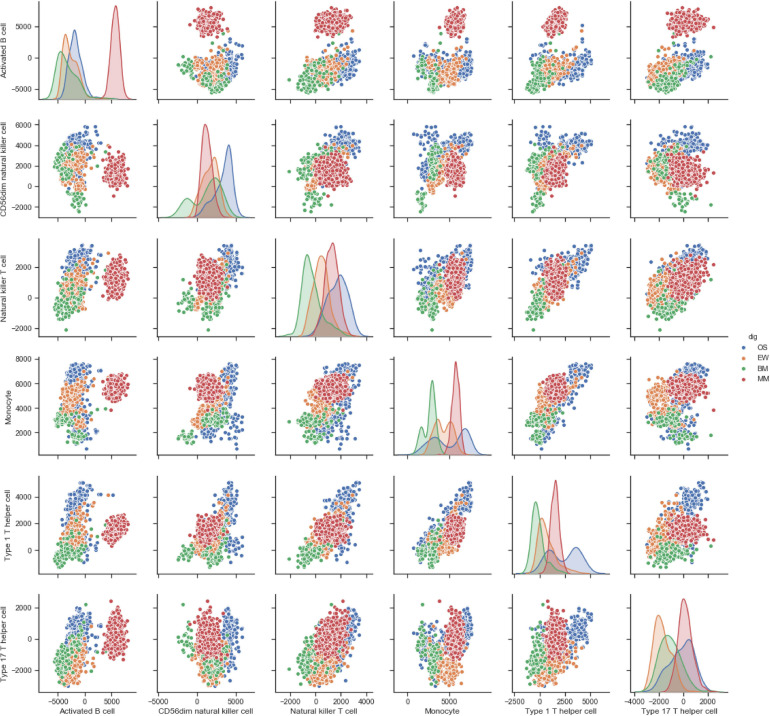
Pair plot of ES score of each cell type across four diseases. The floor area diagram represents ES score distribution across four diseases; the dot plot represents the two-dimensional spatial distribution of each sample. Disease types are annotated by colors. MM, multiple myeloma; EW, Ewing’s sarcoma; BM, prostate cancer bone metastases; OS, osteosarcoma.

Correlation analysis revealed the presence of distinct immune cell correlation signatures for four different diseases (Figure 1C). In OS and EW, there is a general positive correlation between various immune cells. In MM, the immune cells showed weak or no correlation. Meanwhile, the BM correlation matrix reveals a more complex immune cell interaction network. Moreover, the pair plot was drawn based on ES score in each sample, and the result showed that MM can be clearly distinguished from others based on activated B cell and/or immature B cell enrichment score. Additionally, clear BM, OS, and EW clusters can be observed (Figure 2 and Supplementary Figure 1). These results suggest that the four diseases have significant differences in immune microenvironmental infiltration and are potentially susceptible to differential diagnosis by their immune microenvironmental profiles.

### Development of the Diagnostic Model

**FIGURE 3:**
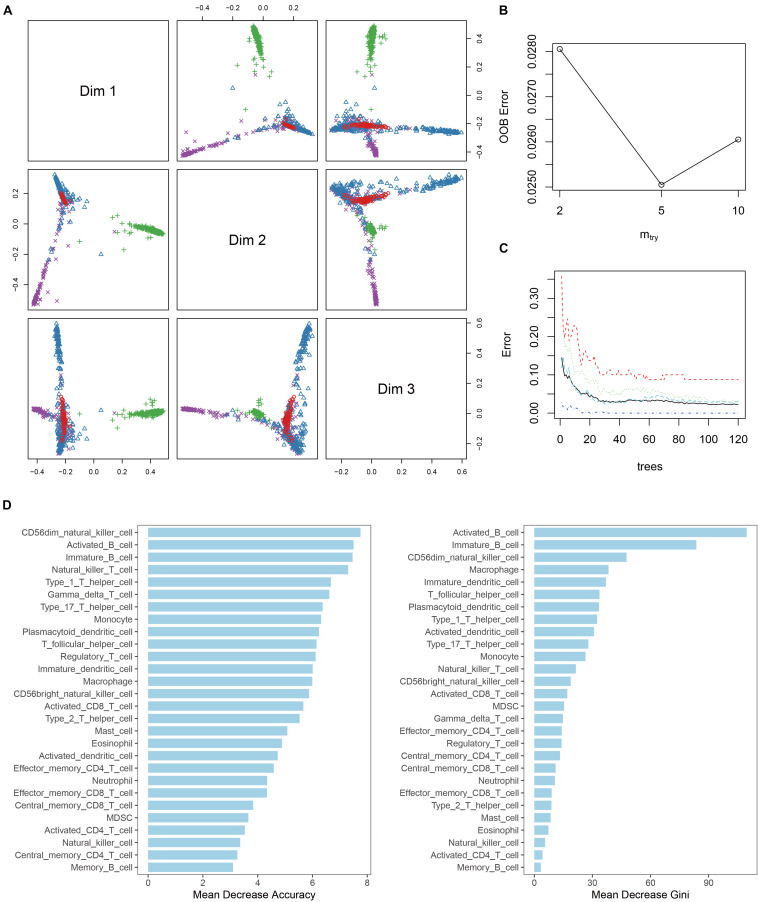
Random forest diagnostic model. **(A)** MDS plot shows the three-dimensional distances between different disease. **(B)** Line plot shows the out-of-bag error (OOB error) of the model with “mtry” parameter setting at 2, 5, and 10. **(C)** Line plot shows the stability of the model with different “tree” parameter settings. **(D)** Histogram of the importance of each ES score in the model.

To develop a multicategorical differential diagnostic model, we used a random forest algorithm to construct the model from a training set of 998 samples. To initially check the feasibility of constructing a classification model, MDS plots were drawn. The result showed that most of the samples can be distinguished base on the immune infiltration profile (Figure 3A). During model tuning, at ntree = 150 and mtry = 5, the diagnostic model had a stable performance with an out-of-bag (OOB) error rate of 2.3% (Figures 3B,C). Continuing to increase the ntree parameter had no significant effect on the diagnostic performance of the model. Meanwhile, increasing the mtry parameter will significantly reduce its accuracy. To further clarify the factors that play a key role in the model, the variable importance histogram was drawn (Figure 3D). Among all 28 immune cell enrichment scores, monocyte, CD56dim NK cell, and activated B cell contributed the most to model accuracy, whereas activated B cell, immature B cell, and CD56dim NK cell contributed the most to sample variance.

### Validation of the Diagnostic Model

**FIGURE 4:**
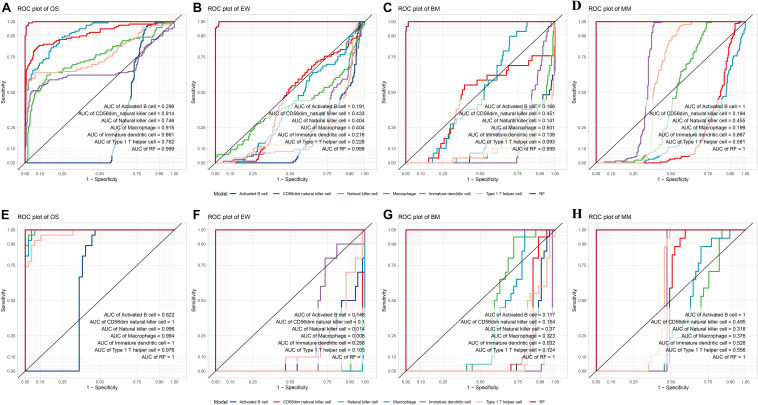
Internal and external validation of the diagnostic model. **(A–D)** ROC plot of the Random forest model compared with a single variable diagnostic model per disease in the test dataset. **(E–H)** ROC plot of the random forest model compared with a single variable diagnostic model per disease in the validation dataset. ROC, receiver operating characteristic; MM, multiple myeloma; EW, Ewing’s sarcoma; BM, prostate cancer bone metastases; OS, osteosarcoma; RF, the random forest diagnostic model.

Internal validation and external validation were carried out, respectively. Firstly, a total of 387 samples in the test dataset were used for internal validation. The overall accuracy of the model was 97.42% (95% CI, 95.3–98.75%, *p* < 2.2^∗^10^–9^), the sensitivity was 0.828, 0.954, 1, and 0.992 on BM, EW, MM, and OS, respectively, and the specificity was 1, 0.997, 0.996, and 0.970 on BM, EW, MM, and OS, respectively. ROC curve showed that the RF model had a significantly better performance compared with single ES score diagnostic models developed with each of the five selected variables (Figures 4A–D). Furthermore, an addition of 74 samples was used for independent external validation (Figures 4E–H). The overall accuracy was 98.65% (95% CI, 92.7–99.97%, *p* < 2.2^∗^10^–16^), with a sensitivity of 1, 1, 0.941, and 1 and a specificity of 1, 0.984, 1, and 1 on BM, EW, MM, and OS, respectively. Table 1 shows the *F*_1_ score, precision, and recall values across all diseases in the training, testing, and validation datasets.

**TABLE 1:** Precision, recall, and *F*_1_ score value of the RF model in the training, testing, and validation datasets.

	OS			EW			MM			BM		
	Training	Testing	Validation	Training	Testing	Validation	Training	Testing	Validation	Training	Testing	Validation
Precision	0.951	0.930	1.000	0.969	1.000	0.909	0.997	1.000	1.000	0.987	1.000	1.000
Recall	0.970	1.000	1.000	0.961	0.954	1.000	1.000	1.000	0.941	0.925	0.828	1.000
*F*_1_ score	0.961	0.964	1.000	0.965	0.976	0.952	0.999	1.000	0.970	0.955	0.906	1.000

### Prognostic Value of Immune Infiltration Score in OS and EW

**FIGURE 5:**
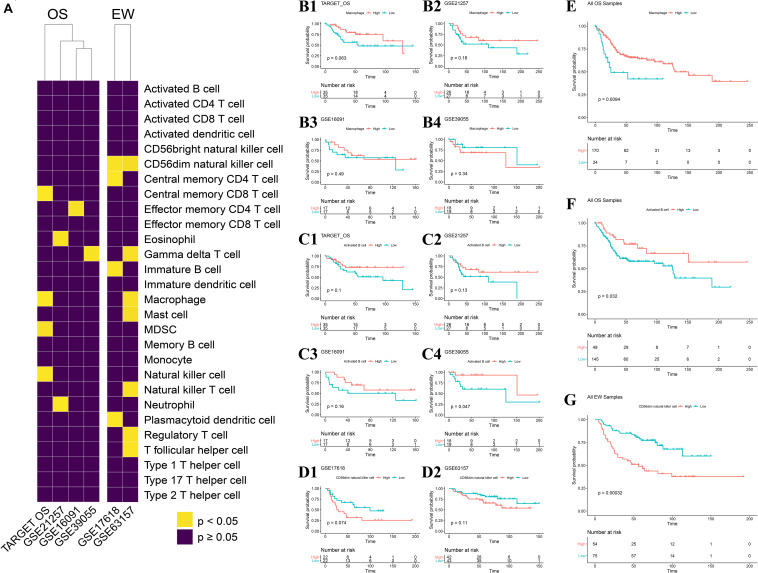
Impact of each immune infiltration score on patients with the four diseases. **(A)** Univariate COX analysis across four OS datasets and two EW datasets; result shown in heatmap, yellow represents *p* < 0.05, violet represents *p* ≥ 0.05. **(B1–B4)** Association of macrophage ES score to OS-specific overall survival in each of the four datasets. **(C1–C4)** Association of activated B cell ES score to OS-specific overall survival in each of the four datasets. **(D1,D2)** Association of CD56dim natural killer cell ES score to EW-specific overall survival in each of the two datasets; median ES score of each cell type per dataset was applied as the cutoff value. **(E)** Association of macrophage ES score to OS-specific overall survival across all samples of the four OS datasets. **(F)** Association of activated B cell ES score to OS-specific overall survival across all samples of the four OS datasets. **(G)** Association of CD56dim natural killer cell ES score to EW-specific overall survival across all samples of the two EW datasets; median ES score of each cell type across all samples in 22 datasets was applied as the cutoff value. OS, osteosarcoma; EW, Ewing’s sarcoma.

Among all 22 datasets, four OS datasets and two EW datasets contain clinical overall survival data. Cox regression analysis was carried out per each dataset. The prognosis value of immune infiltration scores differs across the four OS datasets, as well as between the two EW datasets (Figure 5A). KM analysis was conducted per each dataset using the median ES score as the cutoff value. The result showed that among four OS datasets, macrophage have a similar correlation with patients’ survival (Figures 5B1–B4), as well as activated B cells, yet only activated B cells in dataset GSE39055 has statistical significance (Figures 5C1–C4). A similar result was observed among the two EW datasets (Figures 5D1,D2).

We further combined all datasets per disease and carried out KM analysis using the median ES score of the full sample of the four diseases as the cutoff value, which is more objective and without human intervention. The result indicated that the ES score of both macrophages and activated B cells is positively correlated with OS patient’s survival, with a *p*-value of 0.009 and 0.032, respectively (Figures 5E,F). Also, among the 28 cell types, 17 of them were negatively correlated with EW patients’ survival (Figure 5G and Supplementary Figure 2).

**FIGURE 6:**
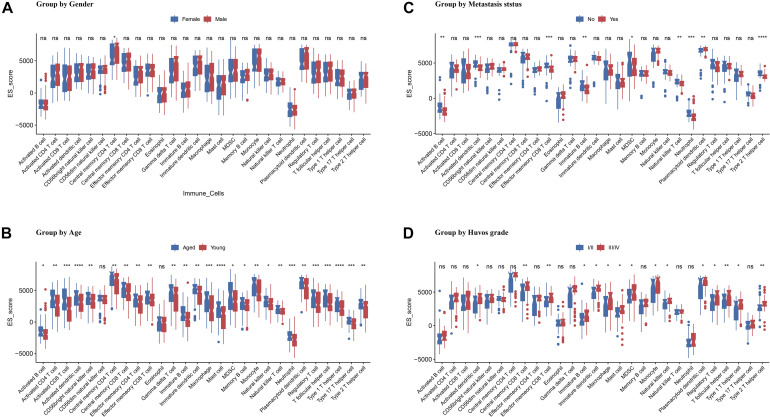
Association of ES score with OS patients’ gender **(A)**, age **(B)**, metastasis status **(C)**, and Huvos grade **(D)**. Asterisk (*) denotes statistical significance examined by Student’s *t*-test. *p < 0.05; **p < 0.01; ***p < 0.001; ****p < 0.0001; “ns” represents *p* > 0.05.

The OS dataset was accompanied by more detailed clinical data. To further clarify the relationship between the immune infiltration score and the clinical characteristics of the patients, we performed *t*-tests for different subgroup conditions. The results showed that there was a difference in CD4T cell infiltration between male and female patients, but no significant difference in the infiltration of other immune cells. In addition, using the age of 18 years as a cutoff, patients were divided into younger and older patients. *t*-test results showed that younger patients generally had lower immune infiltration in 26 of the 28 cell types. Furthermore, when grouped according to tumor metastasis status, the comparison revealed significantly lower ES score of activated B cell, immature B cell, activated DC, effector memory CD8 T cell, MDSC, natural killer T cell, neutrophil, and type 2 T helper cell. Whereas the ES score of the plasmacytoid dendritic cell was significantly elevated. Finally, patients were divided into Huvos grades I/II and III/IV by using tumor necrosis rate of 90% as the cutoff; the results showed more infiltration of CD8 T cell, mast cell, and type 2 T helper cell in patients with high Huvos grade (Figure 6).

## Discussion

Molecular profiling and computer-assisted algorithms are of great significance for fundamental and clinical cancer research (Zhang et al., 2018) and have been widely used for cancer driver gene analysis (Cava et al., 2018), cancer subtyping, and prognosis (Cascione et al., 2013; Junker et al., 2013; Fennell et al., 2019). The immune infiltration microenvironment differs significantly between different malignant tumors, and even in the identical tumor, different immune subtypes exist (Kim et al., 2019). Therefore, the immune infiltration of tumors can potentially be an effective marker for differential diagnosis and prognosis of patients. This study focuses on bone-related malignancies, including OS, EW, MM, and BM; the four diseases have similar tissue conditions, but the origin and progression of these tumors are drastically diverse. Precise differential diagnosis models may be developed based on their immune infiltration profiles. In this study, we obtained 28 immune infiltration scores for each sample across 22 datasets, with batch effect correction and ssGSEA immune infiltration profiling algorithm. The disease diagnosis model was then constructed using Random forest, and evaluated in both internal test datasets and external validation datasets. The association between immune infiltration and patients’ clinical characteristics was evaluated by *t*-test, ANOVA test, and survival analysis. Our results suggested a significantly different immune microenvironment among the four malignancies. In addition, this project developed a diagnostic model with an overall accuracy higher than 97%. On this basis, this study correlated the immune microenvironment with patients’ clinical data and prognosis and found that the highly inflammatory environment in EW was strongly associated with poor prognosis. The effector memory CD8 T cells and type 2 T helper cells in OS were associated with tumor chemotherapy responsiveness and tumor metastasis, while macrophages and activated B cells were closely associated with patient prognosis.

In this study, the overall accuracy of the model based on sample immune infiltration scores was higher than 97%. Studies have shown that ssGSEA is one of the most reliable methods for unsupervised single-sample gene enrichment analysis (Barbie et al., 2009), and the distribution of gene expression ranks inside and outside the gene set. The analysis does not require the entire matrix as input data, and the results are robust (Foroutan et al., 2018). In future applications, the microarray and sequencing data from a single patient can be used for diagnosis. It is an excellent complimentary test and computer-aided diagnostic method.

The immune microenvironment varies significantly among tumors, such as among the four bone-related malignancies included in this study, MM is a malignancy characterized by B cell bone marrow infiltration (Angell et al., 2020), and the proportion and status of B cells in bone marrow aspirate samples can be used to make an accurate diagnosis of the disease. Bone metastatic carcinoma has an extensive immunosuppressive microenvironment, and EW often presents with osteomyelitis-like changes (Kyle et al., 2004; Ottaviani and Jaffe, 2009; Grünewald et al., 2018), accompanied by high expression of inflammatory factors such as IL-6 (Macedo et al., 2017). Finally, OS originates from the bone marrow mesenchyme, and the tumor potentially originates from the monocyte-macrophage lineage (Han et al., 2019), whose immune microenvironment is dominated by monocyte-macrophages and T cells. The results of the present study also revealed that there was a significant enrichment of B cells in MM, and the accurate diagnosis of MM could be made by B cell ratio, with a specificity and sensitivity of 1, which is consistent with the existing literature. Also, in the bone metastatic lesions of prostate cancer, a significant reduction in the infiltration of multiple immune cells, especially T cells, B cells, and DC cells, was observed, consistent with the reported suppressive immune microenvironment of bone metastatic cancer (Fornetti et al., 2018). For OS, its macrophages were most significantly enriched among the four tumors, accompanied by a higher infiltration of T helper cells, mast cells, etc. Notably, unlike the predominant B cells in MM and the significant enrichment of macrophages and CD56dim natural killer cells in OS, the present study did not identify a dominant cell subpopulation in the EW microenvironment, which indicated that a single diagnostic indicator of the immune microenvironment could not be used in the diagnosis of EW.

Tumor immune infiltration is also closely related to patient treatment and prognosis, and the evidence in the literature indicates that increased immune infiltration of M2-like macrophages, CD8 T, NK, mast cells, B cells, and type 2 T helper cells in the EW microenvironment are significantly associated with poor prognosis in EW patients (Kastrinakis et al., 2000; Guzik and Barañska, 2013; Hernandez et al., 2018). Survival analysis of the individual and combined datasets in this study also suggested this result, with increased scores in 17 of 28 immune infiltration scores all suggesting a poor prognosis. Inflammation may play a key role in the progression of EW and is potentially a factor that promotes cancer progression. In addition, the literature indicates a close relationship between OS and myeloid cells, and *in vitro* studies have shown that macrophages present a procancerous role in the progression and evolution of OS, while some clinical studies have shown that activation of monocyte and macrophage infiltration in OS can significantly inhibit OS progression (Liu and Cao, 2016). Adjuvant treatment with macrophage activators such as mifamurtide can improve the survival of patients with highly malignant OS (Jordanov et al., 2009; Huang et al., 2013), while OS patients with high expression of macrophage-related markers such as CD163 had longer overall survival (Durbin et al., 1998). The results of the present study suggest that survival analysis using the median macrophage immune infiltration of each dataset as the cutoff was statistically insignificant and the prognostic trends were divergent. However, by using the median macrophage immune infiltration from four tumors as the cutoff for survival analysis, it was found that patients with low macrophage infiltration had a significantly shorter survival. This result also supports the thesis that macrophages play a protective role in OS. Notably, B cells also suggested significant prognostic value in this study and showed statistically significant differences in both the GSE30699 and combined datasets, suggesting that B cells may have a key role in the prognosis and treatment of OS. B cells are the main component of humoral immunity, and previous research on B cells in OS has been extremely rare. However, in recent years, there has been a spate of breakthroughs in B cell research in oncology. Studies have shown that B cell markers and pathological tertiary lymphoid structure have significant prognostic value (Cabrita et al., 2020) and are important predictors of immunotherapy efficacy (Helmink et al., 2020). Petitprez et al. (2020) identified multiple unique tumor immune subtypes in soft tissue sarcoma and confirmed that B cell infiltration and tertiary lymphoid structures have the strongest correlation with the prognosis of soft tissue sarcoma patients. The present study reveals the prognostic role of B cells in OS at the molecular immune microenvironment level, also suggesting that further studies targeting B cells in OS have a good scientific value. Furthermore, existing studies have shown that age correlates with the level of immune infiltration in patients with OS, and the results of the correlation analysis performed on the integrated dataset in this study are also consistent with this assertion, with younger patients being associated with less immune infiltration (Wu et al., 2020). Moreover, in the present study, effector memory CD8 T cells and type 2 T helper cells were found to be associated with the efficacy of tumor chemotherapy and the development of tumor metastasis. Type 2 T helper cells in OS are still rarely studied; they have long been considered to play a key role in B cell growth, differentiation, and isotype switching and may exert OS suppressive effects in concert with B cells. As for CD8 cells, a study by our colleagues Li et al. (2018) has shown that OS cells can interfere with CD8 T cell infiltration by inhibiting CXCL12 expression and that increasing CXCL12 expression significantly enhances CD8 T cell infiltration and further enhances the eradication of early lung metastases. Although the role of chemotherapy on CD8 T cells is still controversial in academia (Wang et al., 2019; Deng et al., 2020), current studies generally support the anti-tumor effect presented by CD8 T cells. The mechanism by which CD8 T cells, as well as type 2 T helper cells, affect the efficacy and prognosis of chemotherapy in OS still requires further experimental studies.

In conclusion, this paper employed a large dataset of the four tumors for the description of the immune microenvironment of bone-associated tumors and the construction of differential diagnostic models. For the first time, the immune microenvironment differences between the four tumors are analyzed in terms of molecular and molecular-based immune infiltration. The potential of machine learning for disease differential diagnosis was explored. The model showed high accuracy, specificity, and sensitivity. It is a reliable differential diagnosis model with good performance in both the internal test dataset and the external validation dataset and can be used as an auxiliary differential diagnostic tool based on pathological biopsy to further improve the accuracy of diagnosis. The results of the analysis of the integrated dataset are also consistent with many existing published articles and show good scientific validity, meanwhile suggesting that suppressing the highly inflammatory environment of EW and promoting macrophage and B cell infiltration in OS have good potential to be exploited as a therapeutic strategy. The limitation of the present study is that the current diagnostic model cannot be used as a direct substitute for examinations such as clinical pathology; further studies need to be conducted prospectively by collecting samples of the four tumors to further validate the accuracy of the model and advance the application in clinical practice. Also, since the study used publicly available datasets, their clinical data registry was incomplete and therefore unable to compare the changes of immune infiltration in the four tumors of different stages, the association between the immune infiltrative microenvironment of tumors, and the pathological and imaging manifestations of tumor samples also still needs further investigation. Meanwhile, further analysis of tumor-infiltrating cells and immune-related genes and proteins in patients’ biopsy samples by gene expression analysis, flow cytometry, and *in situ* immunohistochemistry is of great importance for the diagnosis and prognosis of the four diseases and the development of novel targeted therapies.

## Materials and Methods

### Gene Expression Data Preprocessing

A total of 22 datasets were included in the study, including OS, EW, BM, and MM samples, as shown in Supplementary Table 1. Microarray gene expression data and clinical information of these datasets were downloaded from the Gene Expression Omnibus (GEO) database^1^ and the Therapeutically Applicable Research to Generate Effective Treatments (TARGET) database^2^. All samples were carefully recurated to avoid errata when using public datasets (Nicolle et al., 2019). The cell line samples, purified tumor cell samples, duplicated samples, samples of normal tissue, and metastasis sample of sites other than bone were excluded from the datasets, only primary bone tumor and cancer bone metastasis samples were kept for further analysis.

Entrez IDs were used to represent genes across different platforms. If multiple probe sets correspond to the same Entrez ID, the one with the highest mean signal was selected as the expression level of the corresponding gene (Miller et al., 2011). The batch effect between different platforms as well as between different datasets in the same platform were adjusted by the ComBat method (Johnson et al., 2007). All expression data were normalized using log2(expression + 1) per each dataset before batch effect removal.

### Immune Infiltration Profiling

The immune signature gene set for 28 immune cell types was obtained from Bindea et al. (2013). The immune infiltration profiling was conducted by the ssGSEA method in R package GSVA (Hänzelmann et al., 2013). Briefly, the gene expression values for a given sample were rank normalized, and an enrichment score was produced using the empirical cumulative distribution functions of the genes in the signature and the remaining genes. This procedure is similar to GSEA, but the list is ranked by absolute expression in each sample. In its final step, within dataset normalization will be carried out routinely to make the results more interpretable. However, since the ssGSEA score normalization within the dataset will introduce unnecessary bias, and study have shown ssGSEA score without normalization is more robust to estimate pathway enrichment (Foroutan et al., 2018), in the present study, ssGSEA scores of 28 immune cell types without normalization (ES score) were used for further analysis.

### Construction of the Diagnostic Model

The random forest method is applied to develop the diagnostic model. Random forest is an ensemble learning method for classification and regression tasks. It operates by selecting random samples from a given dataset, then constructing numerous decision trees during training and voting on each prediction, and finally outputting the class of each tree. It is a widely used classifier for multiple classification tasks and can better cope with sample imbalance and model overfitting.

The datasets were divided into a discovery cohort for model training and testing and an independent external validation cohort for model validation. A total of 1,385 and 74 samples were included in the discovery cohort and validation cohort, respectively. The discovery cohort which contains 421 OS samples, 538 MM samples, 317 EW samples, and 109 BM samples were further divided into a training dataset (*n* = 998) and a testing dataset (*n* = 387) in a 7:3 ratio, by stratified sampling. The model was constructed on the training data using randomForest package in R, with parameters of ntree varying from 10 to 500, and mtry from 0 to 15, meanwhile using parameter tuning function to find out the best combination. The model was then tested for performance on the test dataset and the external validation dataset. ROC curves were visualized using the pROC package.

### Statistical and Survival Analysis

Continuous variables were analyzed using Student’s *t*-tests or ANOVA test using the “stats” package. Four OS datasets (TARGET OS, GSE21257, GSE16091, GSE29055) and two EW dataset (GSE17618, GSE63157) contain the clinical overall survival data, and the prognostic analyses were performed using Kaplan-Meier survival analysis and Cox univariate analyses using the “survival” package. All analyses were carried out using R v.3.6.4. *p* < 0.05 considered being statistically significant. The result was visualized using the “pheatmap” and “survminer” package.

## Conclusion

The random forest diagnostic model based on immune infiltration can accurately perform the differential diagnosis of bone-related malignancies. The immune microenvironment of osteosarcoma and Ewing’s sarcoma has an important impact on patient prognosis. Suppressing the highly inflammatory environment of Ewing’s sarcoma and promoting macrophage and B cell infiltration in osteosarcoma has good potential to be a novel adjuvant treatment option.

## Data Availability Statement

The original contributions presented in the study are included in the article/Supplementary Material, further inquiries can be directed to the corresponding author/s.

## Author Contributions

Z-MY and B-HL design and supervised the study. G-QL collected all datasets and wrote the article with the help of Y-KW and HZ. G-QL, Y-KW, HZ, MA, and W-KZ performed experiments and data visualization. H-YL, YW, L-GJ, and C-YW validated the data. G-QL, L-GJ, and C-YW revised the manuscript. All authors contributed to the article and approved the submitted version.

## Conflict of Interest

The authors declare that the research was conducted in the absence of any commercial or financial relationships that could be construed as a potential conflict of interest.

## Footnotes

1 http://www.ncbi.nlm.nih.gov/geo/

2 https://ocg.cancer.gov/programs/target/
