# Supplementary figures and images for: Application of Immune Infiltration Signature and Machine Learning Model in the Differential Diagnosis and Prognosis of Bone-Related Malignancies
Source: Front Cell Dev Biol. 2021 Apr 15;9:630355. doi: 10.3389/fcell.2021.630355 (PMC8082117; doi:10.3389/fcell.2021.630355)

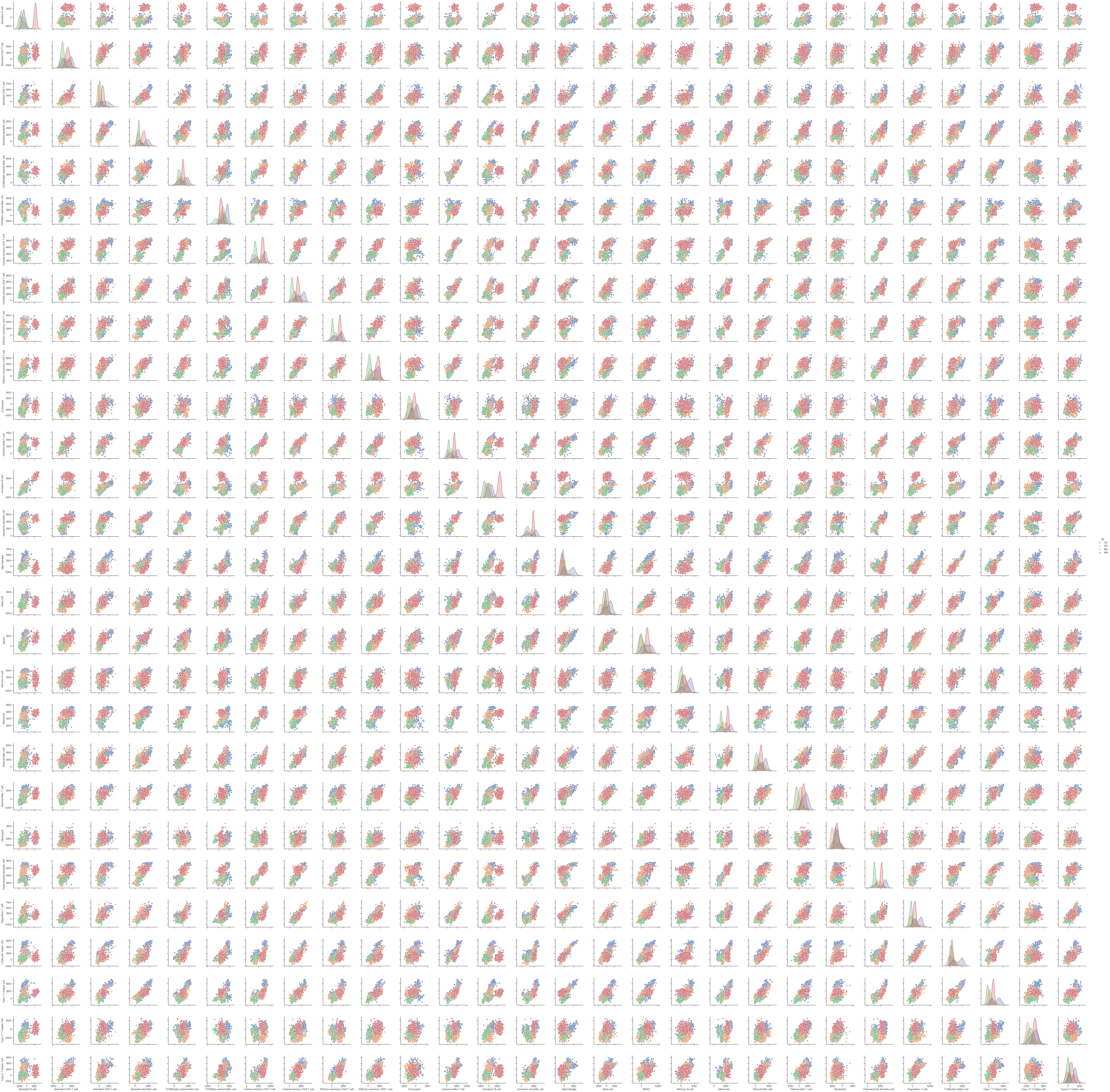

Supplement: Supplementary file 1 [file Image_1.JPEG]

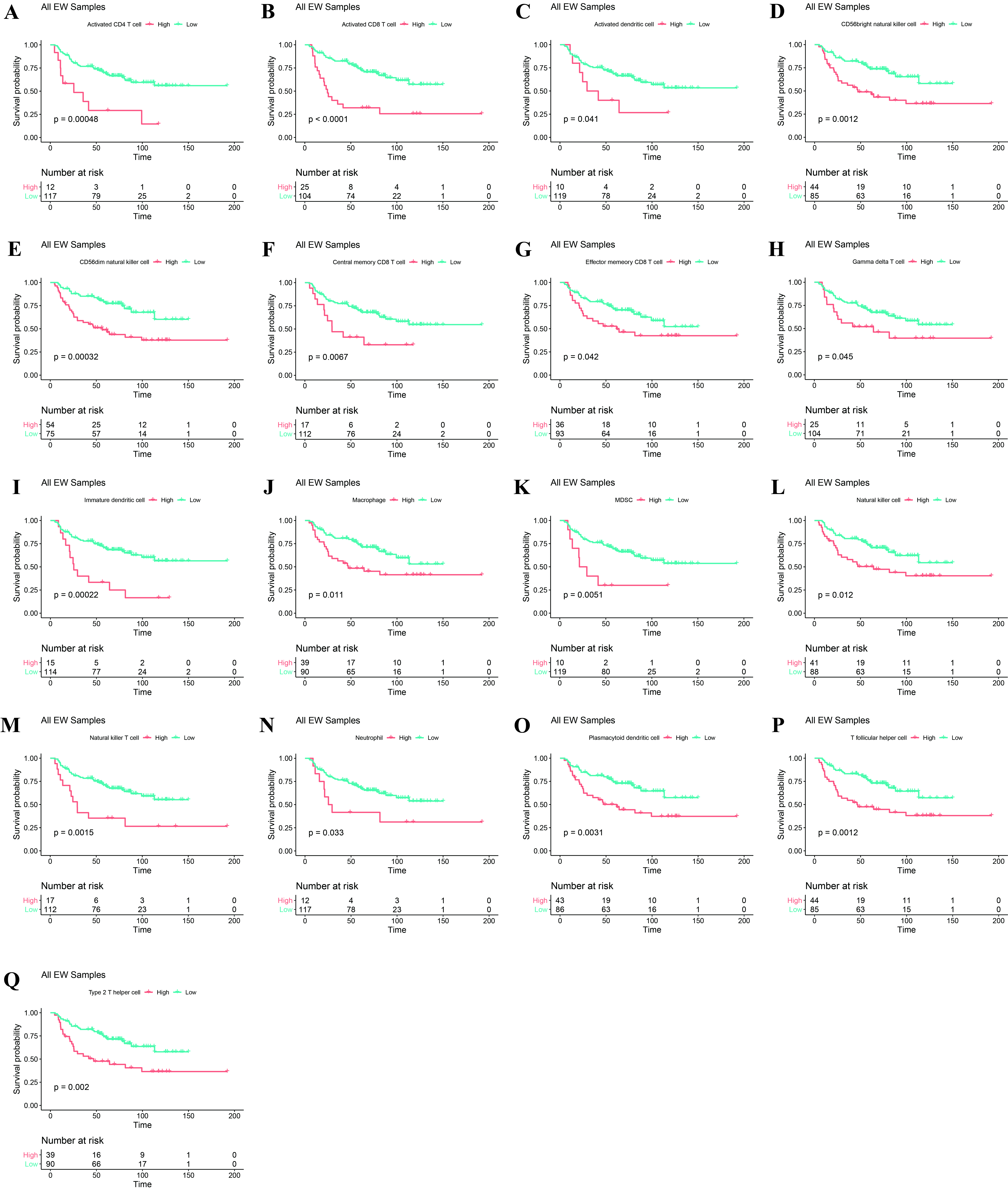

Supplement: Supplementary file 2 [file Image_2.JPEG]
